# A Methodology for Assessing Tumor Clonality of Adult T Cell Leukemia/Lymphoma

**DOI:** 10.1016/j.omtm.2020.10.015

**Published:** 2020-10-22

**Authors:** Tomohiro Yamakawa, Naoki Uno, Daisuke Sasaki, Norihito Kaku, Kei Sakamoto, Kosuke Kosai, Hiroo Hasegawa, Yasushi Miyazaki, Katsunori Yanagihara

**Affiliations:** 1 Department of Laboratory Medicine, Nagasaki University Graduate School of Biomedical Sciences, Nagasaki 852-8501, Japan; 2 Department of Hematology, Atomic Bomb Disease and Hibakusya Medicine Unit, Atomic Bomb Disease Institute, Nagasaki University, Nagasaki 852-8523, Japan

## Abstract

While clonal heterogeneity has been demonstrated in most cancers, quantitative assessment of individual tumor clones has not been translated to inform clinical practice. A few methods have been developed to investigate the tumor clonality of adult T cell leukemia/lymphoma (ATLL), but currently there is no clinically translatable method available for quantifying individual tumor clones in ATLL patients. Here, we present a methodology to assess the tumor clonality of ATLL and quantify patient-specific tumor clones in a clinical setting. The methodology consists of three steps: (1) selective amplification of restriction fragments containing a human T cell leukemia virus type 1 (HTLV-1) integration site, (2) amplicon deep sequencing to estimate the clonal structure and identify HTLV-1 integration sites of dominant clones, and (3) digital PCR targeting the HTLV-1 integration sites of the dominant clones to quantify specific tumor clones. We successfully tracked individual tumor clones using this approach and demonstrated that each clone had a distinct response to therapies. The procedure is straightforward and clinically feasible, which should facilitate the proper assessment and management of ATLL.

## Graphical Abstract

**Figure:**
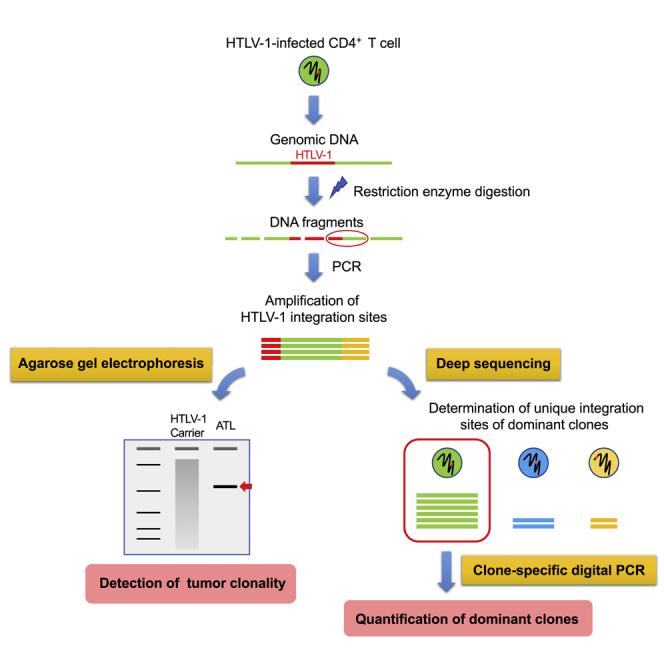

## Teaser

A novel procedure is presented for detecting tumor clonality and quantifying individual tumor clones in adult T cell leukemia/lymphoma, which could facilitate the monitoring of patient-specific tumor clones in clinical practice.

## Introduction

Recent advances in sequencing technologies have uncovered tumor heterogeneity in most cancers.^1^ Distinct subpopulations of tumor cells display remarkable variability in response to treatments.^2^ Increasing evidence of functional diversity between tumor subpopulations suggests the need for assessing clonal heterogeneity in clinical practice.^1^^,^^3^ However, inference of tumor composition using DNA sequencing data is challenging^4^ and clinically applicable methods for assessing individual tumor clones have yet to be developed.

Adult T cell leukemia/lymphoma (ATLL) is a hematological malignancy caused by clonal proliferation of CD4^+^ T cells infected with human T cell leukemia virus type 1 (HTLV-1). Following HTLV-1 infection, viral DNA is synthesized from viral RNA by viral reverse transcriptase and integrated into the human genome. The integrated viral DNA is called a provirus. Each infected cell contains a unique integration site (UIS) because integration occurs throughout in the human genome,^5^ and the majority of CD4^+^ T cells from peripheral blood of HTLV-1-infected individuals contain a single copy of a provirus.^6^ Therefore, an infected cell lineage, which refers to an ancestral infected cell and its descendants, can be identified by a UIS. Most individuals infected with HTLV-1 are asymptomatic and never develop ATLL. However, in approximately 5% of asymptomatic HTLV-1 carriers, infected cells proliferate clonally and cause ATLL after a latency period of several decades.^7^ The clonality of ATLL can be determined based on the UIS because when an ancestral infected cell proliferates clonally and gives rise to tumor cells, the UIS of the infected cell lineage becomes the signature of that tumor clone. While gene rearrangements of T cell receptor and immunoglobulins have been used to define the clonality of lymphoproliferative neoplasms, UISs remain the standard target for clonality detection in ATLL because tumor cells only arise from T cells infected with HTLV-1.

Although UISs can be determined by whole-genome sequencing (WGS),^5^ a few groups have developed methods to identify UISs by amplicon deep sequencing and then used these UISs to analyze the clonality of ATLL.^8^, ^9^, ^10^ One group reported that, in 89% of cases, ATLL arose from a single ancestral infected cell, while in 11%, it arose from two.^11^ These methods are based on enrichment of provirus-containing sonicated DNA fragments prior to sequencing.^8^, ^9^, ^10^ While such methods are able to reveal the tumor clonality of ATLL without WGS, they are not feasible in a clinical setting because both the sequencing data analysis and the experimental procedures are very demanding. A previous study reported a simple method based on the amplification of UISs in restriction-enzyme-digested genomic DNA to investigate the clonality of HTLV-1-infected individuals,^12^ but has not been developed for clinical use.

Assessment of individual tumor clones helps physicians make appropriate disease assessments and decide on the optimal therapy.^13^ Herein, we present a clinically practical methodology for identifying and evaluating individual tumor clones in ATLL patients.

## Results

### Experimental Design for Selective Amplification of Restriction Fragments Containing HTLV-1 Provirus

**Figure 1:**
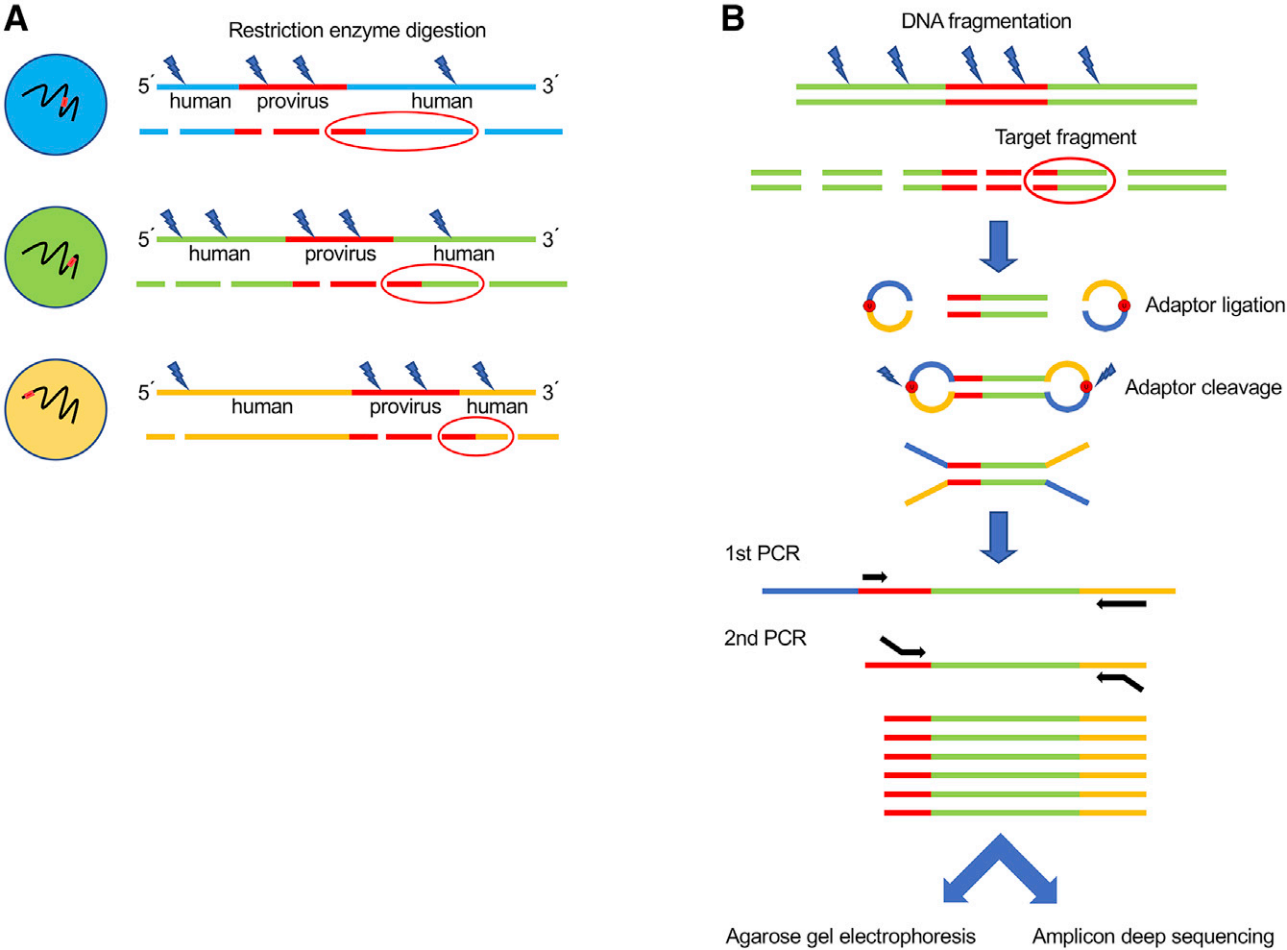
Schematic of Amplification of Restriction Fragments that Contain a HTLV-1 Integration Site (A) The size of restriction fragments that contain both provirus and human sequences is unique to each infected cell. (B) The fragment containing the 3′ LTR of a provirus and human sequences was amplified by semi-nested PCR. Following fragmentation of genomic DNA by a restriction enzyme, adaptors were ligated to the restriction fragments and subsequently cleaved. The first round PCR was performed using forward and reverse primers that bind to the 3′ LTR of a provirus (red) and an adaptor sequence (yellow), respectively. The second PCR was performed using a different primer set. The forward primer used in the second PCR was designed to bind slightly inside of the binding site of the first forward primer. The binding sites of the reverse primers used in the first and second PCR overlap at their 3′ ends. Both forward and reverse primers used in the second PCR have 5′ sequences added for the downstream sequencing analysis. Oligonucleotide sequences used for the semi-nested PCR are shown in Table S2. The products were analyzed by electrophoresis and deep sequencing.

We designed the experiments to investigate HTLV-1-infected cell lineages based on UISs. A DNA library was prepared using a restriction enzyme instead of sonication, because the size of restriction fragments containing both provirus and human sequences is unique to each infected cell lineage (Figure 1A). HpyCH4V was selected as the restriction enzyme because the fragments it generates from digestion of the human genome would be expected to be short enough to be amplified by PCR. Following DNA fragmentation, we amplified fragments that contained a HTLV-1 integration site by semi-nested PCR. Specifically, we ligated adaptors to restriction fragments using a DNA library preparation kit and used the fragment library as the template for PCR. The PCR was performed to amplify fragments that contained a UIS next to the provirus 3′ long terminal repeat (LTR), which is well conserved, whereas the 5′ LTR is often truncated.^14^ We carried out PCR using a forward primer binding to the provirus 3′ LTR and a reverse primer binding to an adaptor sequence (Figure 1B). The fragments containing the provirus 3′ LTR were further enriched by a second PCR, using a nested forward primer that was placed internal to the first forward primer. Of note, the size of the PCR product would still be unique to each infected cell lineage, because although the viral sequence length is the same in all amplicons, the length of the human sequence varies according to the restriction site.

### Clonal Proliferation of HTLV-1-Infected Cells Can Be Assessed by Agarose Gel Electrophoresis

**Figure 2:**
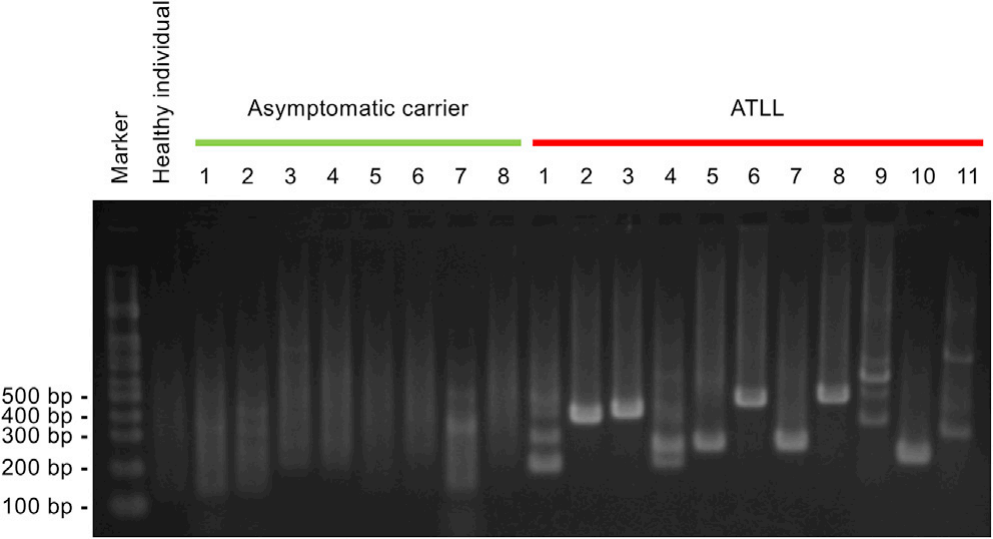
Clonal Proliferation of HTLV-1-Infected Cells Can Be Detected by Agarose Gel Electrophoresis Genomic DNA was extracted from PBMCs from asymptomatic HTLV-1 carriers (n = 8), ATLL patients (n = 10), and a healthy individual, or for ATLL case 4 from a lymph node sample, and digested with HpyCH4V. Restriction fragments containing both the 3′ LTR of a provirus and human sequences were amplified as depicted in Figure 1B and analyzed by agarose gel electrophoresis.

We extracted genomic DNA from peripheral blood mononuclear cells (PBMCs) from asymptomatic HTLV-1 carriers (n = 8), ATLL patients (n = 10) and a healthy individual, and for ATLL case 4, from a resected lymph node. The genomic DNA was digested with HpyCH4V and adaptors were ligated to the restriction fragments to prepare a restriction fragment library containing adaptor sequences. We ensured that the size of most fragments of the library were <1 kb without size selection (Figure S1). We subsequently performed semi-nested PCR as described above and analyzed the products by agarose gel electrophoresis (Figure 2).

Smeared bands were observed in samples derived from asymptomatic carriers, suggesting that restriction fragments containing a provirus are present but vary in size. In other words, smeared bands suggest the presence of small amounts of various HTLV-1-infected cells in the samples. Clear bands were observed in samples from all ATLL patients. A band indicates a large amount of a specific restriction fragment that contains a provirus; that is, a band represents clonal proliferation of an infected cell. A single band was observed in seven patients (cases 2, 3, 5, 6, 7, 8, and 10), indicating that an infected cell proliferated clonally to become a monoclonal tumor population. More than one band was observed in four patients (cases 1, 4, 9, and 11), suggesting that more than one infected cell proliferated clonally to produce the oligoclonal tumor populations. Thus, the electrophoresis results addressed the clinically important questions of whether HTLV-1-infected cells proliferate clonally and, if so, are they monoclonal or oligoclonal.

### Clonal Structure Constructed by Amplicon Deep Sequencing

**Figure 3:**
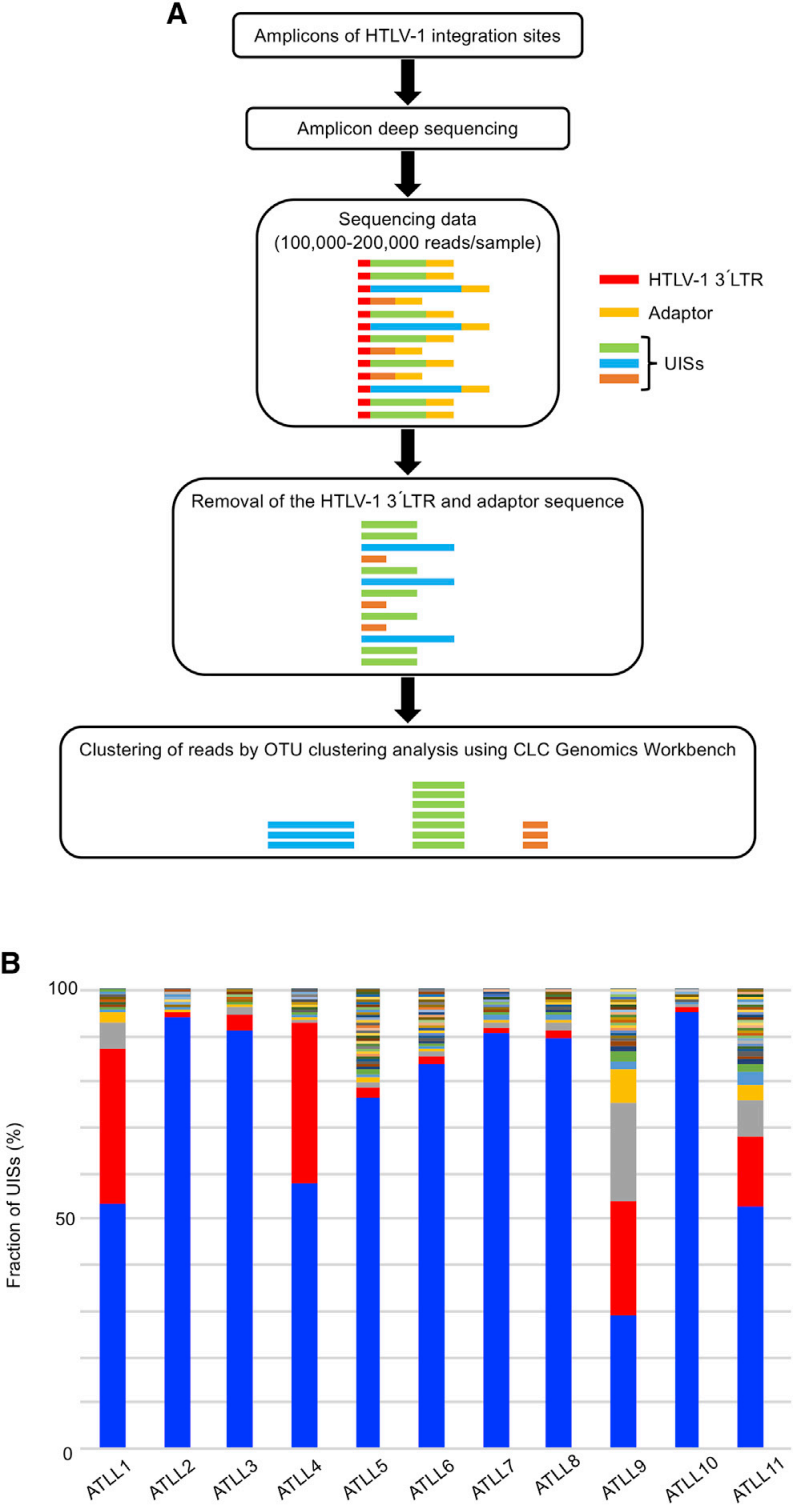
Clonal Structure of ATLL Patients Analyzed by Amplicon Deep Sequencing Based on HTLV-1 Integration Sites (A) Sequencing data analysis of amplicons that contain the 3′ LTR of a provirus, human sequences, and the adaptor sequence. Both provirus and adaptor sequences were trimmed and only human sequences were clustered. (B) The proportions of UISs in ATLL patients. Restriction fragments containing both the 3′ LTR of a provirus and human sequences were amplified as depicted in Figure 1B and subjected to deep sequencing. All samples were PBMCs except for case 4, which was an involved lymph node.

We next assessed and quantified the UIS composition of the ATLL samples to determine the clonal structure of each patient’s malignant clone(s). We performed deep sequencing of the semi-nested PCR products to determine UISs in individual infected cells and quantified the fraction they comprised. We acquired 100,000–200,000 reads in depth, determined the UISs, clustered them, and constructed clone structures therefrom (Figure 3A). Seven patients (cases 2, 3, 5, 6, 7, 8, and 10) had only one dominant clone, whereas in four patients (cases 1, 4, 9, and 11), more than one clone was found (Figure 3B). These results were consistent with the appearance of the semi-nested PCR products after electrophoresis (Figure 2).

### Quantification of Individual Dominant Clones by Clone-Specific digital PCR

**Figure 4:**
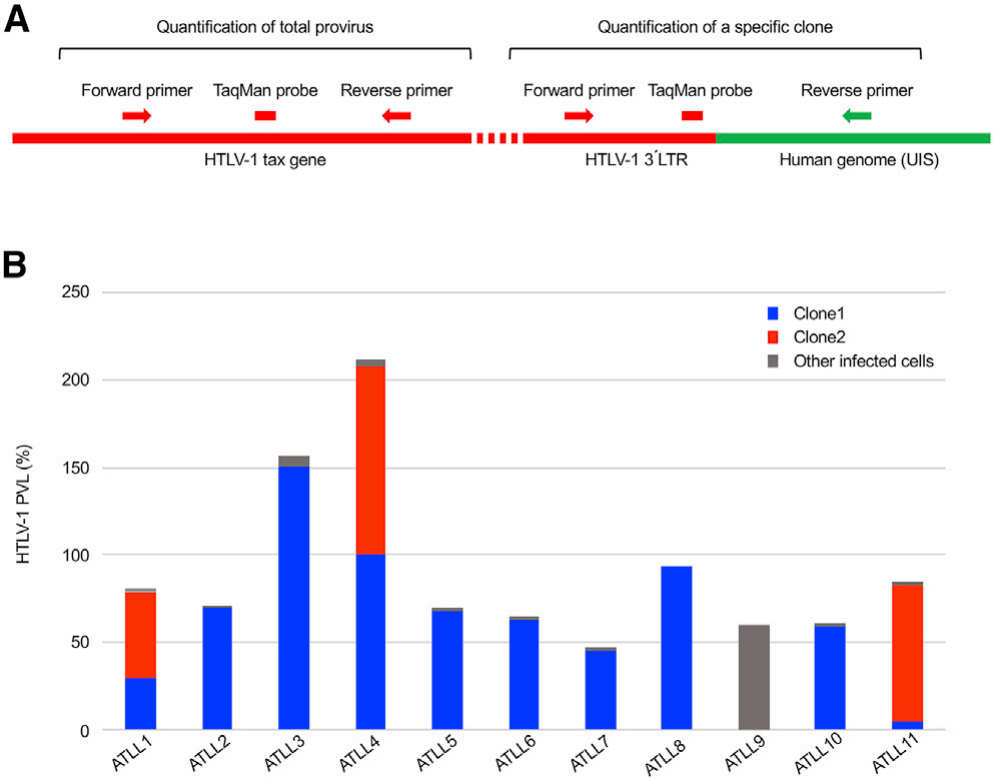
Quantification of Patient-Specific Tumor Clones by CS-dPCR (A) Design of TaqMan probes and primers to quantify specific clones and total infected cells. The reverse primer binding sites were placed in UISs to quantify specific clones. Oligonucleotide sequences are provided in Table S2. (B) The PVLs of specific clones, as well as that of total infected cells, were measured by dPCR. The samples were the same as in Figure 3B. The blue and red clones of each patient are identical to those shown in Figure 3B. Subtraction of blue and red clones from total infected cells are shown as other infected cells in gray.

To validate the clonal structure estimated by amplicon deep sequencing, we next measured the proviral load (PVL) of dominant clones, as well as that of total infected cells. We developed clone-specific digital PCR (CS-dPCR) to quantify individual dominant clones using a forward primer binding within the provirus 3′ LTR, a TaqMan probe binding the 3′ LTR downstream of that, and a reverse primer binding to the UIS (Figure 4A). We also designed another TaqMan probe and primer set within the HTLV-1 *tax* gene to target provirus alone and enable quantification of the PVL of total infected cells (Figure 4A). We performed dPCR using the same genomic DNA templates used in amplicon deep sequencing and quantified the copy number of dominant clones, as well as that of total infected cells. The PVL was determined as described in the Materials and Methods and indicates the proportion of HTLV-1-infected cells in a sample unless, as may occur rarely, more than one provirus is present in an infected cell.

We quantified the dominant clone, shown in blue in Figure 3B, in patients who contained only one dominant clone (cases 2, 3, 5, 6, 7, 8, and 10). In each case, the clone represented the majority of the infected cells (Figure 4B). Two clones, which are shown in blue and red in Figure 3B, were quantified in those cases with two major clones (cases 1, 4, and 11). The majority of infected cells consisted of the two clones (Figure 4B), although the ratios of the two clones were not always consistent with those estimated by deep sequencing. Three clones, which are shown in blue, red, and gray in Figure 3B, were quantified in case 9. However, none of them were dominant (Figure 4B). These results demonstrated that the clonal structure determined by amplicon deep sequencing was not always accurate but enabled the UISs of dominant clones in 10 out of 11 patients to be identified.

### Clone Dynamics in Response to Treatments in ATLL Patients

**Figure 5:**
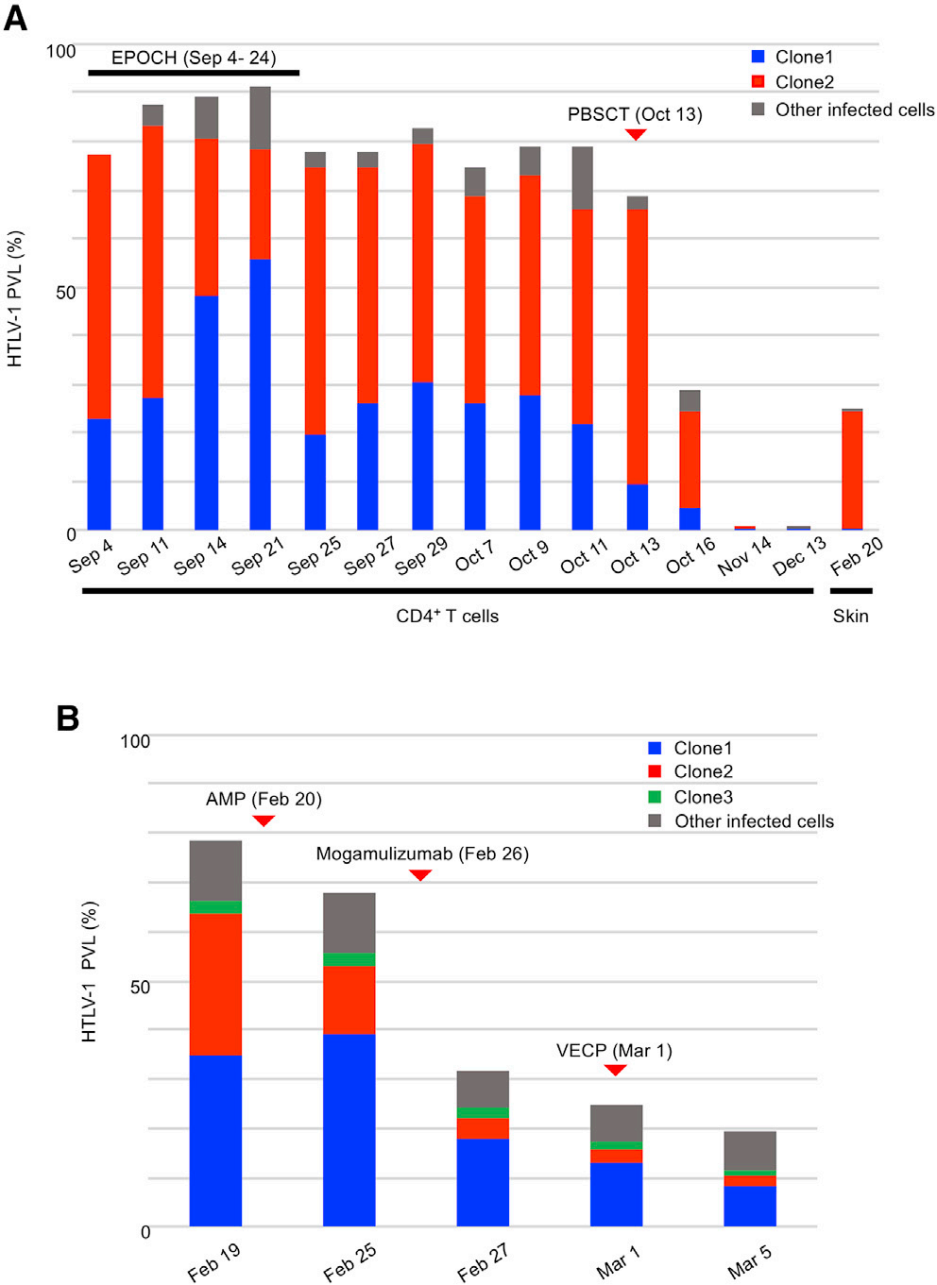
Clonal Tracking in ATLL Patients Who Had Two Abundant Clones The PVLs of patient-specific clones, as well as that of total infected cells among CD4^+^ T cells were tracked by dPCR in ATLL cases 1 and 11. The blue and red clones of each patient are identical to those shown in Figures 3B and 4B. Subtraction of tracked clones from total infected cells are shown as other infected cells in gray. (A) The PVLs of two clones, as well as that of total infected cells, were tracked during EPOCH chemotherapy and after PBSCT in case 1. All samples were peripheral blood CD4^+^ T cells, except for the last, which was a skin lesion. (B) The PVLs of three clones as well as that of total infected cells were tracked in case 11, who received mogamulizumab in addition to the VCAP-AMP-VECP regimen, which included vincristine, cyclophosphamide, doxorubicin, prednisone, ranimustine, vindesine, etoposide, and carboplatin.

We next applied CS-dPCR to track individual clones in two patients with two clones (cases 1 and 11) and monitored the response to treatment. We measured the PVL of the two clones, as well as that of total infected cells in case 1 (Figure 5A). Clone 1 increased during EPOCH (etoposide, cyclophosphamide, vincristine, doxorubicin, and prednisone) chemotherapy and decreased after its completion, whereas clone 2 decreased during the chemotherapy and increased afterward. Both clones and other infected cells were almost eliminated from the peripheral blood after peripheral blood stem cell transplantation (PBSCT). However, a skin lesion developed 4 months after the PBSCT. We extracted genomic DNA from the skin lesion, carried out CS-dPCR, and found that the skin infiltrate was of clone 2 origin.

We tracked three clones in case 11 (Figure 5B). The patient was treated with mogamulizumab in addition to combination chemotherapy. We measured the PVL of the three clones, as well as that of total infected cells for 2 weeks. After chemotherapy with AMP (doxorubicin, ranimustine, and prednisone), clone 2 decreased but clone 1 increased suggesting that this therapy was more effective against clone 2 than clone 1. Both clones decreased following mogamulizumab, and clone 1 then further decreased after therapy with VECP (vindesine, etoposide, carboplatin, and prednisone). Little change was observed in clone 3 after each therapy. The PVL of total infected cells decreased after each of the three therapies.

The clonal structure thus changed markedly during treatment and CS-dPCR allowed us to assess the progression and resistance of each clone in response to treatment quantitatively. By contrast, the total PVL, which is currently used clinically as a measurement of PVL, does not provide any information about clone dynamics and could, therefore, lead to misunderstanding of the disease progression and response to therapy.

## Discussion

We have developed a comprehensive methodology for clonal assessment of HTLV-1-infected cells (Figure S2). From a clinical point of view, our methodology has substantial advantages over other methods. First, it allows us to address whether and, if so, how many, HTLV-1-infected cells proliferate clonally using PCR followed by agarose gel electrophoresis without the need for sequencing. Southern blot hybridization is currently used for this purpose in clinical laboratories, but requires a lot of genomic DNA, time, and labor; our method overcomes these disadvantages and could potentially replace it. Second, UISs of dominant clones can be identified by deep sequencing of PCR products followed by clustering sequencing reads using user-friendly interfaces without any bioinformatics analysis. The analysis of sequencing data is straightforward and less complicated than previous methods, which rely on sonicated fragment libraries being amplified and sequenced. Such methods require computational analysis to reconstruct the clone structure because sequencing reads are different in size even if they contain a common integration site. Sequencing data should be interpreted by computational methods that analyze UISs to identify dominant clones.^8^, ^9^, ^10^ The pipelines leading to the analysis of sequencing data are often complicated and unstandardized,^4^ whereas our use of a restriction enzyme instead of sonication for DNA fragmentation avoids these problems. Third, once UISs of dominant clones are identified by amplicon deep sequencing, CS-dPCR can be designed to enable unique and specific clones to be quantified and tracked per patient. Currently, there is no means to quantify patient-specific clones in a clinical setting. We developed CS-dPCR to quantify and track patient-specific clones and to provide a clinically useful tool for clonal assessment of ATLL. Adoption of our methods by clinical laboratories is feasible and could contribute toward optimal tailored care for individual patients with ATLL. The present methodology is distinct from that of a recently published method that amplifies HTLV-1 integration sites using linear amplification-mediated PCR.^15^ This method estimates clonality using sanger sequencing but can neither determine clonal proliferation of infected cells nor estimate clonality by using electrophoresis prior to sequencing. Moreover, it is not designed to track individual tumor clones.

The total PVL was over 100% in two patients (cases 3 and 4; Figure 4B). There are two possible explanations for this. One is that a provirus has integrated into more than one genomic region in an infected cell. If an infected cell that harbors two distinct UISs proliferates clonally and leads to development of monoclonal tumor population, two abundant UISs should be identified at the same frequency because every tumor cell contains the two UISs. While this is clinically uncommon,^11^ it is conceivable in case 4, as two abundant UISs were identified at the same frequency (Figure 4B). The other possible explanation is amplification of a provirus in an infected cell. Amplification of genomic regions often occurs in cancer, including ATLL.^5^ If a provirus is amplified in an infected cell during clonal proliferation of the cell, more than one provirus is present per cell and thus the PVL can be over 100%. This could explain the finding in case 3 as only one dominant UIS was identified (Figure 4B).

We found three limitations in the methodology. First, the absence and presence of HTLV-1 provirus were barely distinguishable by electrophoresis; a faint smeared band was observed even in the PBMCs derived from the healthy individual (Figure 2). The nonspecific amplification could be due to reverse primer binding because every template fragment contains the adaptor sequence to which they bind. The reverse primers that are extended from every fragment could lead to nonspecific amplification if forward primers do not bind to a provirus. Second, distinct bands may be detectable by electrophoresis even in asymptomatic carriers when the total PVL is very low; a few faint bands were visible in the sample from the asymptomatic carrier 7 whose PVL was less than 0.5% (Figure 2; Table S1). When the size of the infected cell population is very small, clonal composition tends to be less heterogeneous and therefore the intensity of several bands may be increased. Third, we failed to identify UISs of dominant clones in case 9. This is probably due to the failure to amplify restriction fragments containing UISs of the dominant clones. The UISs of dominant clones should be identified by amplicon deep sequencing as long as restriction fragments that contain the UISs of the dominant clones are amplified by PCR. However, if the 3′ end of the provirus of the dominant clone is truncated, or if the restriction fragment is too long to be amplified by PCR,^16^ restriction fragments containing UISs cannot be amplified. The truncation of the 3′ end of a provirus in ATLL cells is uncommon but could occur in rare cases.^14^ Our method, like some others, cannot identify UISs of dominant clones in such cases.

## Materials and Methods

### Clinical Samples

Peripheral blood samples were taken from a healthy individual who was not infected with HTLV-1, asymptomatic individuals who were infected with HTLV-1 (asymptomatic carriers; n = 8), and ATLL patients (n = 10). For ATLL case 4 with lymphomatous disease, an involved inguinal lymph node sample was used rather than a blood sample, and for ATLL case 1, an involved skin tissue was used in addition to a blood sample. Clinical and demographic characteristics of the asymptomatic carriers and ATLL patients are provided in Table S1. All samples were collected at Nagasaki University Hospital. The study was approved by the ethics committee of Nagasaki University Hospital. PBMCs and CD4^+^ T cells were separated from whole blood using Lymphoprep (Axis-Shield Density Gradient Media), and an EasySep Direct Human CD4^+^ T Cell Isolation Kit (STEMCELL Technologies), respectively.

### Preparation of Restriction Fragment Library

Genomic DNA was extracted from PBMCs, CD4^+^ T cells, and the lymph node and skin samples using a QIAamp DNA Blood Mini Kit (QIAGEN). Genomic DNA (1 μg) was fragmented using the restriction enzyme HpyCH4V (New England Biolabs) at 37°C for 1 h. The enzyme was subsequently inactivated at 65°C for 20 min. NEBNext adaptors were ligated to the fragments using a NEBNext Ultra DNA Library Prep Kit for Illumina (New England Biolabs) and NEBNext Mulitiplex Oligos for Illumina (New England Biolabs). NEBNext End Prep, Adaptor Ligation, and Cleanup without Size Selection steps were performed according to the manufacturer’s protocol. The “PCR Enrichment of Adaptor Ligated DNA” step of the protocol was not performed; instead, semi-nested PCR was carried out as described below.

### Semi-Nested PCR

The first round PCR was performed in 50 μL reaction mixture containing 5 μL adaptor ligated DNA fragments, 25 μL NEBNext Q5 Hot Start HiFi PCR Master Mix, and 0.5 μM forward and reverse primers. The product of the first amplification reaction was diluted in water (1:100) and used as the template for the second PCR. The second round of PCR was performed in 50 μL reaction mixture containing 5 μL diluted template, 25 μL NEBNext Q5 Hot Start HiFi PCR Master Mix, and 0.5 μM forward and reverse primers. The cycling conditions for both first and second round PCRs were 98°C for 30 s, 35 cycles of 98°C for 10 s, and 72°C for 75 s, followed by 72°C for 2 min and an infinite hold at 4°C. The primer sequences are provided in Table S2.

### Agarose Gel Electrophoresis

The semi-nested PCR product was mixed with loading buffer (Wako) and loaded into wells of a 2% agarose gel alongside a 100 bp DNA Ladder RTU (Gene DireX) size marker. After electrophoresis, the agarose gel was stained with ethidium bromide and DNA revealed with UV light.

### Amplicon Deep Sequencing

The semi-nested PCR products were purified using Agencourt AMPure XP (Beckman Coulter) according to the manufacturer’s instructions. The purified amplicons were quantified using a Bioanalyzer High Sensitivity DNA Analysis (Agilent) system, diluted with water to 18 pM, and mixed with Ion PGM Hi-Q View Ion Sphere Particles (ISPs) using an Ion PGM Hi-Q View OT2 Kit (Thermo Fisher Scientific) in the Ion OneTouch 2 System (Thermo Fisher Scientific). The template-positive Ion PGM Hi-Q View ISPs were prepared according to the Ion PGM Hi-Q View OT2 Kit - 400 protocol and sequenced using an Ion PGM Hi-Q View Sequencing Kit (Thermo Fisher Scientific) and Ion 318 Chip Kit v2 BC (Thermo Fisher Scientific). Sequencing data were analyzed using CLC Genomics Workbench (CLC Bio). The HTLV-1 3′ LTR and the adaptor sequence were trimmed from the amplicon sequence and UISs were clustered by operational taxonomical unit (OTU) clustering analysis and the UISs of dominant clones were identified. The amplicons generated from the HTLV-1 5′ LTR that contained only viral sequences were removed before clustering analysis.

### Digital PCR

Digital PCR (dPCR) was performed using a QuantStudio 3D Digital PCR System (Applied Biosystems) in 14.5 μL reaction mixture containing 2 ng/μL genomic DNA template, 1× QuantStudio 3D Digital PCR Master Mix v2, 0.45 μM forward and reverse primers, 0.125 μM TaqMan probe, and 1× TaqMan copy number reference assay RNase P (Applied Biosystems). Sequences of probes and primers are shown in Table S2. The reporter dyes of the TaqMan probes hybridizing to provirus and RNase P were FAM and VIC, respectively. The cycling conditions were 96°C for 10 min, 39 cycles of 98°C for 30 s, and 60°C for 2 min, 60°C for 2 min, and infinite hold at 10°C. The PVL was calculated as: PVL = 100 × (provirus copy number × 2)/RNase P copy number.

## Supplemental Information

Document S1. Tables S1 and S2 and Figures S1 and S2

Document S2. Article plus Supplemental Information
