# Supplementary material for: A Methodology for Assessing Tumor Clonality of Adult T Cell Leukemia/Lymphoma
Source: Mol Ther Methods Clin Dev. 2020 Oct 22;19:467–73. doi: 10.1016/j.omtm.2020.10.015 (PMC7701009; doi:10.1016/j.omtm.2020.10.015)
Supplement: Document S1. Tables S1 and S2 and Figures S1 and S2 [file mmc1.pdf]

OMTM, Volume *19*

Supplemental Information

A Methodology for Assessing Tumor Clonality of Adult T Cell Leukemia/Lymphoma

Tomohiro Yamakawa, Naoki Uno, Daisuke Sasaki, Norihito Kaku, Kei Sakamoto, Kosuke Kosai, Hiroo Hasegawa, Yasushi Miyazaki, and Katsunori Yanagihara

Table S1. Demographic characteristics of patients

Patient ID	Age	Sex	Diagnosis		Total PVL (%)[1)]
AC1	79	F	Asymptomatic carrier		8.7
AC2	68	F			14.8
AC3	56	F			6.1
AC4	72	M			9.8
AC5	66	F			2.9
AC6	40	F			12
AC7	72	M			<0.5
AC8	65	M			54.5
ATLL1	58	M	ATLL	Acute	48.6
ATLL2	64	M		Chronic	49.4
ATLL3	83	F		Acute	124.3
ATLL4	75	M		Lymphomatous	176.1
ATLL5	59	M		Chronic	56.9
ATLL6	64	M		Acute	61.7
ATLL7	83	M		Acute	17.1
ATLL8	75	F		Chronic	90.3
ATLL9	73	F		Smoldering	56.8
ATLL10	60	F		Chronic	60.9
ATLL11	68	M		Chronic	27.1

[1)] Total PVL in PBMCs was quantified by real-time PCR.

Table S2. Oligonucleotide sequences

Semi-nested PCR		Target	5´ - 3´
1st PCR	Forward primer	HTLV-1 3' LTR	CCTTTCATTCACGACTGACTGCCG
	Reverse primer	Adaptor	CAAGCAGAAGACGGCATACGAGATCGTGATGTGACTGGAGTTCAGACGTGTGCTCTTCCGATC
2nd PCR[1)]	Forward primer	HTLV-1 3' LTR	CCATCTCATCCCTGCGTGTCTCCGACTCAGTTACAACCTCGATTGGCTCGGAGCCAGCGACAGCCCAT
			CCATCTCATCCCTGCGTGTCTCCGACTCAGAACCATCCGCGATTGGCTCGGAGCCAGCGACAGCCCAT
			CCATCTCATCCCTGCGTGTCTCCGACTCAGATCCGGAATCGATTGGCTCGGAGCCAGCGACAGCCCAT
			CCATCTCATCCCTGCGTGTCTCCGACTCAGTCGACCACTCGATTGGCTCGGAGCCAGCGACAGCCCAT
			CCATCTCATCCCTGCGTGTCTCCGACTCAGCGAGGTTATCGATTGGCTCGGAGCCAGCGACAGCCCAT
			CCATCTCATCCCTGCGTGTCTCCGACTCAGTCCAAGCTGCGATTGGCTCGGAGCCAGCGACAGCCCAT
			CCATCTCATCCCTGCGTGTCTCCGACTCAGTCTTACACACGATTGGCTCGGAGCCAGCGACAGCCCAT
			CCATCTCATCCCTGCGTGTCTCCGACTCAGTTCTCATTGAACGATTGGCTCGGAGCCAGCGACAGCCCAT
			CCATCTCATCCCTGCGTGTCTCCGACTCAGTCGCATCGTTCGATTGGCTCGGAGCCAGCGACAGCCCAT
			CCATCTCATCCCTGCGTGTCTCCGACTCAGTAAGCCATTGTCGATTGGCTCGGAGCCAGCGACAGCCCAT
	Reverse primer	Adaptor	CCTCTCTATGGGCAGTCGGTGATAGTTCAGACGTGTGCTCTTCCGATC-s-T[2)]

[1)] Blue and red sequences are sequencing primer and Tag sequences, respectively.

[2)] -s- indicates phosphorothioate bond.

CS-dPCR	Target	Patient	Patient-specific clone	5´ - 3´
Forward primer	HTLV-1 3' LTR			CCTTTCATTCACGACTGACTGC
TaqMan probe	HTLV-1 3' LTR			[FAM]GAGCGACAGCCCATCCTATAGCACTCTCC[BHQ1]
Reverse primer	UIS	ATLL1	clone 1	ATCTCCAGAGTATAAATGTTCTCCCT
			clone 2	GGAGATAGTGACAAGAGTGAAACTC
		ATLL2	clone 1	GCAGCTTCACAGCAACATCTG
		ATLL3	clone 1	CATTTGTGCCATGGGGGAAG
		ATLL4	clone 1	TTGTGGTGTGCTGTTCCACA
			clone 2	GATCGATCGTGCTCTTCCGATC
		ATLL5	clone 1	GGTCCTGTCCTGCCAACCTC
		ATLL6	clone 1	CAGGCACCCGAATGGGATTA
		ATLL7	clone 1	GGTAGATCAATGGGACAAAGTAACA
		ATLL8	clone 1	TCATGGCCACATGGAAAAGTG
		ATLL9	clone 1	TGCTTTGTTTTGGCTGTCTGC
			clone 2	GTCTGGGTCCCTCCCTTCA
			clone 3	AACCGAATCCAGCAGCACAT
		ATLL10	clone 1	CACACTGGCATGTATTTGGAGTTT
		ATLL11	clone 1	TCTCTCAGGAGAAAGCAAGGA
			clone 2	TCACATCACTTCTCAGATCCTCA
			clone 3	TTGAGCCCAGGAGTTCAAGT

dPCR for total PVL	Target	5´ - 3´
Forward primer	HTLV-1 tax	CCCACTTCCCAGGGTTTGGA
TaqMan probe	HTLV-1 tax	[FAM]CCAGTCTACGTGTTTGGAGACTGTGTACA[BHQ1]
Reverse primer	HTLV-1 tax	GGCCAGTAGGGCGTGA

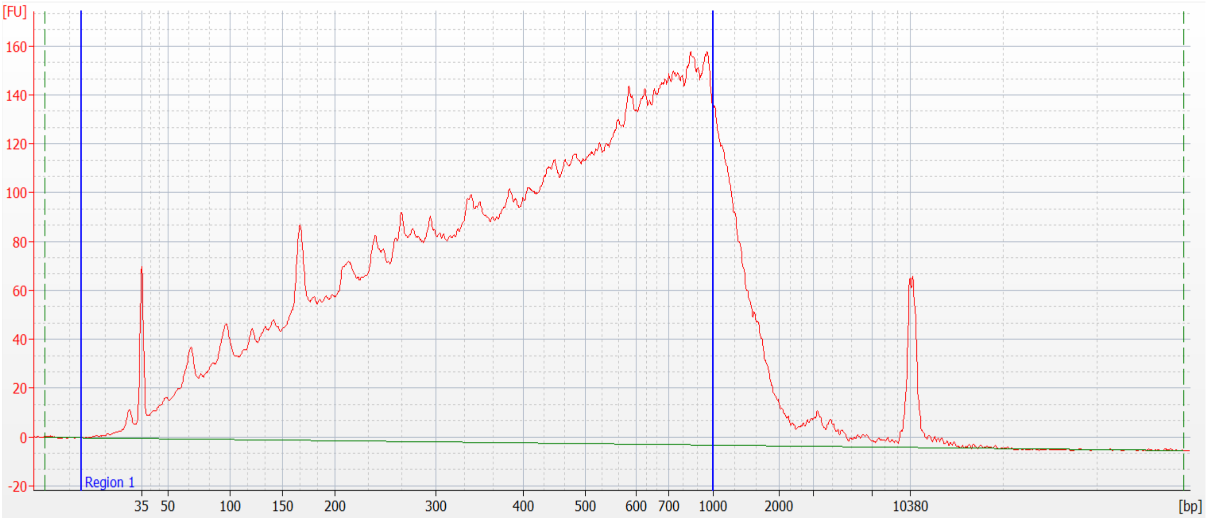

Figure S1. Size distribution of DNA fragments after digestion with HpyCH4V. Genomic DNA of a healthy individual was digested with HpyCH4V and the size of fragmented DNA was analyzed by Bioanalyzer.

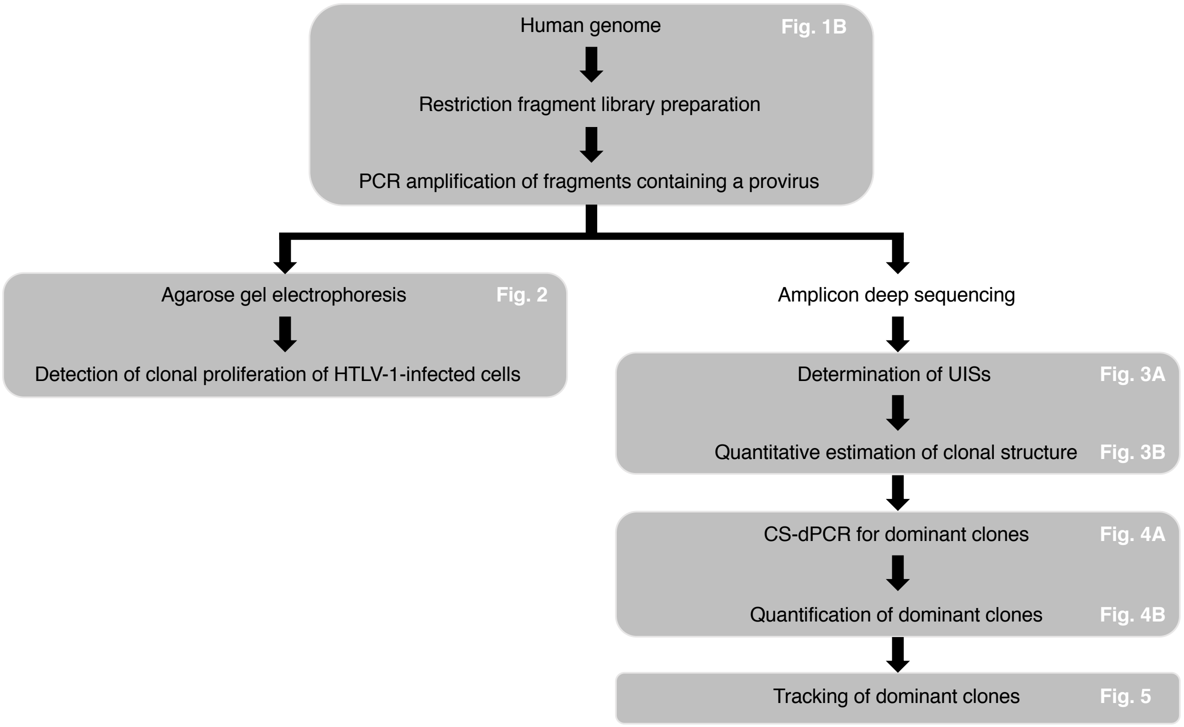

Figure S2. Experimental workflow.
